# Electrophysiology Meets Printed Electronics: The Beginning of a Beautiful Friendship

**DOI:** 10.3389/fnins.2018.00992

**Published:** 2019-01-04

**Authors:** Lilah Inzelberg, Yael Hanein

**Affiliations:** ^1^Sagol School of Neuroscience, Tel Aviv University, Tel Aviv, Israel; ^2^School of Electrical Engineering, Tel Aviv University, Tel Aviv, Israel

**Keywords:** EMG, EEG, printed electrodes, skin electronics, wearable sensors

## Abstract

Electroencephalography (EEG) and surface electromyography (sEMG) are notoriously cumbersome technologies. A typical setup may involve bulky electrodes, dangling wires, and a large amplifier unit. Adapting these technologies to numerous applications has been accordingly fairly limited. Thanks to the availability of printed electronics, it is now possible to effectively simplify these techniques. Elegant electrode arrays with unprecedented performances can be readily produced, eliminating the need to handle multiple electrodes and wires. Specifically, in this Perspective paper, we focus on the advantages of electrodes printed on soft films as manifested in signal transmission at the electrode-skin interface, electrode-skin stability, and user convenience during electrode placement while achieving prolonged use. Customizing electrode array designs and implementing blind source separation methods can also improve recording resolution, reduce variability between individuals and minimize signal cross-talk between nearby electrodes. Finally, we outline several important applications in the field of neuroscience and how each can benefit from the convergence of electrophysiology and printed electronics.

## Introduction

### Surface Electrophysiology

In recent years, electroencephalography (EEG) and surface electromyography (sEMG) have been suggested for countless new applications, such as brain-machine interfaces (BMIs), neurological and psychiatric diagnostics, bio-feedback, rehabilitation, sports, and emotion detection (Griss et al., 2002; Chowdhury et al., 2013; Hwang et al., 2013; Khan and Hong, 2017). Efforts were directed toward improved signal processing methods (Hyvärinen, 2011; Farina and Holobar, 2016; Naik et al., 2016) low noise electronics and low-impedance electrodes (Sun et al., 2012; Yeo et al., 2013; Piervirgili et al., 2014; Wang et al., 2017). Until recently, electrode technology achieved relatively little progress. Most contemporary skin electrodes rely on conducting gels, a lingering bottleneck in skin electrophysiology. Gels and adhesive pastes may cause skin irritation and short circuits between adjacent electrodes.

Furthermore, signal quality gradually degrades over time as the gel dries (Searle and Kirkup, 2000; Kappenman and Luck, 2010). As an alternative, dry skin-penetration electrodes shaped as pins and barbs were suggested, utilizing micro electro mechanical systems (MEMS) techniques. Improving the electrode skin interface with applied pressure on the electrode was also explored (Searle and Kirkup, 2000; Griss et al., 2001; Liao et al., 2011; Lopez-Gordo et al., 2014). These technologies are nevertheless cumbersome and do not provide a true solution for prolonged use.

Overall, despite the clear motivation to use EEG and sEMG in many clinical and consumer settings, contemporary technology is too cumbersome for non-laboratory use. State of the art systems suffer from major technological challenges including: (a) Poor electrode quality manifested primarily by signal-to-noise ratio (SNR) reduction over time; (b) signal cross-talk between nearby electrodes resulting in limited source identification; and (c) low resolution recording (Searle and Kirkup, 2000; De Luca et al., 2010; Fiedler et al., 2014; Hug and Tucker, 2016).

### Printed Electronics

Printed electronics is a key technology in a wide range of applications including displays and touch screens (Berggren et al., 2007). The growing need for flexible electronics has also contributed to vast expertise in printing materials that can accommodate bending and stretching. Printing of flexible electronics addresses the needs of huge markets, a fact which has driven its rapid development and maturation (Kamyshny and Magdassi, 2014; Sabatini et al., 2017). This resulted in the development of affordable and high performing inks, substrates, printing methods and tools, lamination processes, and more (Jiang et al., 2016). Substantial academic research is still carried out in this field, although well-established techniques along with vast expertise are already available in the industry for small to medium and large volume production.

Biocompatible materials, suitable for long-term use in direct contact with the skin, are also available commercially. Materials that can stretch to accommodate significant skin deformation during movement were developed (Jeong et al., 2013; Matsuhisa et al., 2017). Resolution achieved in printing techniques is limited compared with micro-fabrication, (Kim et al., 2011; Webb et al., 2013; Yeo et al., 2013; Madhvapathy et al., 2018) yet it is perfectly compatible with the needs of skin electrophysiology while benefiting from significantly reduced prototyping and production costs.

### Electrophysiology Meets Printed Electronics

**FIGURE 1:**
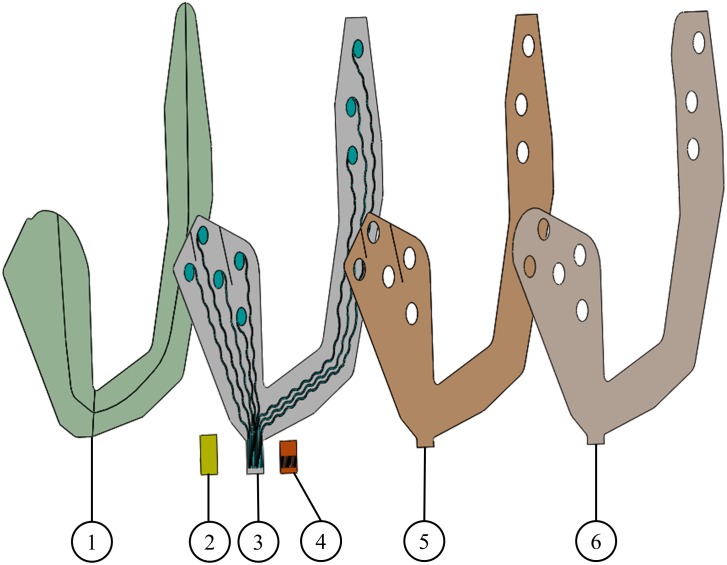
Screen printed electrodes. (1) Support layer, (2) stiffener, (3) conductive ink printed on a soft substrate, (4) Kapton, (5) skin adhesive layer, and (6) release layer. Layers 1 and 6 are temporary and are used to mechanically stabilize the electrode array before its placement on the skin.

Printing electrode arrays on flexible substrates, such as polyimide or polyester, was suggested as a mean to improve user convenience and electrode placement (on hair-free regions). Printed electrodes using various conductive inks, such as thermoplastic silver ink (Myllymaa et al., 2013a), carbon (Bareket et al., 2016; Inzelberg et al., 2018a) and poly(3,4-ethylenedioxythiophene, PEDOT) (Inzelberg et al., 2018a) were applied recently in several applications (Figure 1). Printed EEG electrode arrays, such as *BrainStatus* (Lepola et al., 2014; Miettinen et al., 2018) and *cEEGGrid* (Debener et al., 2015) opened new opportunities in neurological investigations and diagnostics. Optimal material selection for printing the electrodes also permit safe artifact-free magnetic resonance imaging (MRI) and computerized tomography (CT) scanning (Myllymaa et al., 2013a,b). In the realm of sEMG, several studies demonstrated the benefits of printing (Scalisi et al., 2015; Zucca et al., 2015; Bareket et al., 2016; Ferrari et al., 2018; Inzelberg et al., 2018a). Ultrathin sEMG electrodes can maximize the contact area, lower the contact impedance, while reducing movement artifacts (Ferrari et al., 2018; Inzelberg et al., 2018a). High density printed sEMG emerges as a non-invasive method to acquire precise information of muscle activation by increasing the electrode number and enabling data analysis schemes (Marco, 2015; Scalisi et al., 2015).

## Soft Printed Electrode Arrays and Improved Signal Quality

Ideally, electrophysiological electrodes should be capable of prolonged recording time, should be flexible and stretchable to conform with the human skin, should provide user convenience and achieve high-resolution recording with no cross-talk. The mechanical properties of the ink have to satisfy two main conditions: durability in bending and stretching, and high flexibility to achieve conformity with the skin. In our investigations we have addressed this issue by identifying inks that can sustain extreme folding without breaking. This proved to be particularly important for larger EEG electrodes. The electrical conductivity of the ink is important in EEG applications when long traces resistance may reach values comparable with the skin-electrode impedance. In this case, silver traces are needed. Detailed discussion regarding ink and material properties is available in Inzelberg et al., 2018a.

Early printed arrays, used to date, were realized on polyimide or polyester. Although flexible, they are not soft enough to conform and stretch with the human skin, and as such do not resolve the need for a conducting gel. However, printing the electrodes on soft substrates, such as polyurethane, facilitated a dramatic improvement (beyond user convenience achieved with the polyimide or polyester electrodes). Principally, a conducting gel is no longer needed to establish good electrode-skin conductivity. Secondly, recording quickly stabilizes and remains stable over time. Along with these two benefits, data analysis can be used to reduce cross-talk and inter- and intra-subject variability. Moreover, soft printed electrodes allow high-resolution and array customization.

**FIGURE 2:**
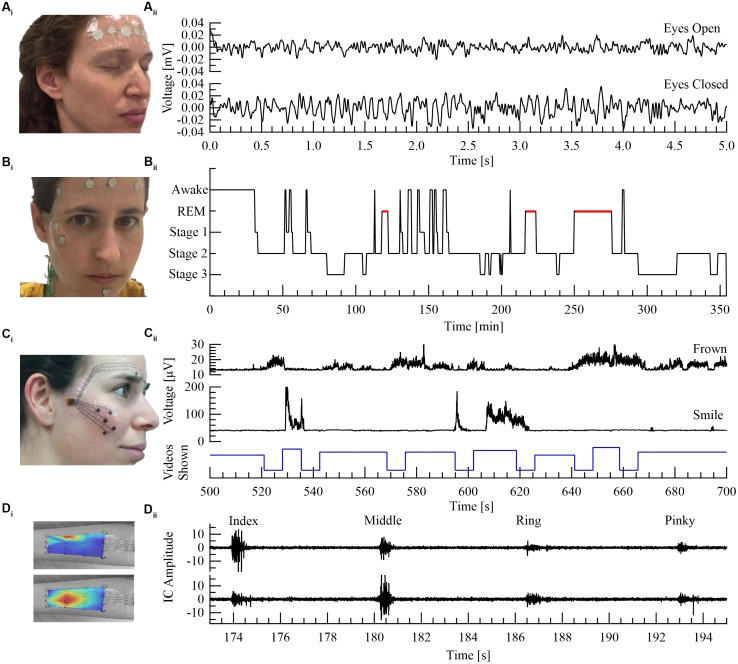
Four printed electrode arrays customized for different neurological or psychological applications: **(A)** EEG monitoring. **(B)** Sleep stage monitoring (adapted from Shustak et al., in press). **(C)** Facial expressions as a marker of neuro-psychiatric conditions (adapted from Inzelberg et al., 2018a). **(D)** Limb muscle activation. **(A_i_)** Electrode array located at the forehead region. **(A_ii_)** Voltage versus time of EEG signals showing clear alpha waves (differentiating between eyes open and closed). **(B_i_)** Electrode array for sleep stage monitoring with four electrodes located at the forehead (EEG), two next to the right eye (EOG) and two by the chin region (sEMG). **(B_ii_)** Sleep hypnogram during 6 h. **(C_i_)** Electrodes array to capture muscle activity close to the eyebrow and cheek regions. **(C_ii_)** Root Mean Square (RMS) sEMG signals during video watching (frowning at the top panel, smiling at the middle panel and the stimuli trace at the bottom panel). **(D_i_)** 16 channel electrode array located at the forearm with superimposed color maps of calculated independent components (ICs) shown in **(D_ii_)**. Red color in IC maps specifies maximal muscle activation. **(D_ii_)** IC signals, generated from fastICA algorithm, show clear separation of middle finger versus index finger activations. Written informed consent was obtained from all the individuals presented in this image.

Commercially available gelled electrodes are relatively rigid and thus can record effectively from relatively flat skin areas. Their recording performances from uneven or curved skin regions (such as the face) are limited. Moreover, as the skin-electrode impedance of a gelled electrode tends to deteriorate over time, the recording quality degrades over time (Searle and Kirkup, 2000). Dry electrodes, usually in the form of stiff metal pads, do not require gel. However, they lack flexibility resulting in poor adhesion to the skin, especially in curved areas (Searle and Kirkup, 2000; Fiedler et al., 2014). Electrode arrays printed on a highly soft support, on the other hand, facilitate skin-electrode conformity at uneven skin surfaces. As soft printed electrodes are dry, they enable stable, prolonged recordings. While commercial dry electrodes interfere with user comfort, electrodes printed on highly soft substrates allow superior user convenience with minimal attention to the experimental setup (Fiedler et al., 2014; Zucca et al., 2015; Ferrari et al., 2018; Inzelberg et al., 2018a,b). This property allows prolonged recording of several hours in sEMG (Bareket et al., 2016; Inzelberg et al., 2018b) and EEG (Figure 2B; Shustak et al., in press).

Cross-talk, defined as the contamination of the sEMG signal by the myoelectric activity in adjacent muscles, is a major challenge in skin electrophysiology (Chowdhury et al., 2013; Hug and Tucker, 2016). It may cause a change in the sEMG signal shape depending on electrode location, and in many cases, a muscle can be falsely identified as generating electrical activity when it does not (Hug and Tucker, 2016). Cross-talk depends strongly on anatomical and physiological parameters. Reducing the electrode size and precisely controlling the inter-electrode distance is a direct approach to reduce cross-talk (Chowdhury et al., 2013). Nevertheless, reduced electrode dimension is beneficial as long as electrodes can record distinguishable signals, which are not located in high-density muscle regions. A more relevant approach to address cross-talk in sEMG is by applying mathematical methodologies, such as blind source separation (BSS) (Hyvärinen et al., 2001; Farina et al., 2004; Naik et al., 2008, 2016; Farina and Holobar, 2016). As printed electrodes can be designed to include high-density arrays with multiple recording sites, they are ideal for source identification. This analysis cannot be achieved with contemporary electrodes due to their relative large size, limited density, and cumbersome placement. In facial expression mapping in particular, fast independent component analysis (fastICA) algorithm is a powerful tool (Inzelberg et al., 2018b). Once fastICA is applied on the data, the relative weight at each recording site is calculated and the independent components for each event are obtained. This methodology reduces variability between repetitions and individuals as it does not rely on electrode specific location (Hyvärinen, 2011; Inzelberg et al., 2018b).

In Figure 2 we show four examples of temporary-tattoo electrode arrays designed for four different uses: (1) Sub hairlines EEG; (2) EEG, Electrooculography (EOG) and sEMG array for sleep stage [stages 1–3 and Rapid Eye Movement (REM)] recording (six hr); (3) sEMG from the cheek and eyebrow regions to detect emotional affect; and (4) High resolution (16) electrode array for sEMG of the forearm.

## Novel Applications

As summarized above, printed electrodes on soft substrates offer several important benefits over conventional electrode technology. Precise electrode placement is an important part in skin electrophysiology, commonly necessitating experienced technicians, and prolonging the placement process. Replacing such a manual procedure by a quick placement of a single adhesive patch has a clear advantage in reducing time and manpower costs. Printing enables customization of electrode arrays to ideally fit specific individuals, needs and applications. The large number of electrodes integrated in a single printed array provides high-resolution recordings with reduced motion artifacts, compared with conventional electrodes (Inzelberg et al., 2018b).

Reports from the last decades addressed numerous applications of skin electrophysiology. Most of these reports were based on gel electrodes. Clearly, the number of applications of wearable electrophysiological sensors is immense. We review below several examples of applications we currently study, emphasizing the benefits of printed electronic technology for neurological applications, while discussing existing knowledge gaps for each application.

### Sleep Monitoring in Neurological Evaluation

Sleep disturbances appear in many neurological diseases and are one of the most common non-motor symptoms in Parkinson’s disease (PD) (Paparrigopoulos, 2005; Postuma, 2014). REM sleep behavior disorder (RBD) is a form of parasomnia characterized by the loss of normal skeletal muscle atonia during REM sleep, with prominent motor activity accompanying dreaming. In the search for markers of the disease, RBD holds much promise, as it is highly specific for phenoconversion to PD (Postuma et al., 2013). The gold standard for diagnosis of sleep disorders is based on studies conducted overnight in a laboratory setting using polysomnography (PSG). However, since RBD is episodic in nature, and does not necessarily appear every night, it is difficult to diagnose in a single night. In addition, this method is expensive, is not feasible for widespread clinical practice, and often does not reflect everyday life conditions. An electrode array with the capability to record EEG, EOG, and sEMG signals over multiple nights in the home environment holds great promise.

We recently designed, implemented and tested such a wireless dry electrode system for sleep stage analysis. sEMG, EOG, and EEG were successfully recorded using a wireless system. Stable recordings were achieved both in a hospital environment and in a home setting (Shustak et al., in press). Utilizing our wireless recording technology (Inzelberg et al., 2017, 2018b) with a printed EEG electrode array, enabled clear separation between open versus closed eyes (Figure 2A). Sleep monitoring during a six hr session showed clear differentiation of sleep stages (Figure 2B).

### Facial Expressions as a Marker of Neuro-Psychiatric Conditions

Precise mapping (spatial and temporal) of facial expressions provides a vast promise in medical assessment because many neurological and psychiatric disorders are typified by abnormal activation patterns. One example is the clinical feature of PD, hypomimia; the reduction or loss of spontaneous facial expressions (Argaud et al., 2016; Bologna et al., 2016). On the other hand, an example demonstrating excessive facial muscle activation is Tourette syndrome (TS), that includes involuntary fast, brief and repetitive facial movements in the form of tics (Brandt et al., 2016; Muth, 2017). These examples point to the necessity to objectively classify facial expressions for diagnostic and therapeutic purposes.

We recently demonstrated a unique ability to differentiate between facial muscle activations using printed electrode arrays (Inzelberg et al., 2018b). Furthermore, the ability to record spontaneous, commanded and mimicry facial expressions, utilizing both screen- and inkjet-printed electrodes comprising both the cheek and the eyebrows regions in a single integrated array, was also implemented (Figure 2C; Inzelberg et al., 2018a).

### Limb Muscle Activation in Neurology

Abnormal limb muscle activation reflects a multitude of pathological conditions that encompass diseases of the muscles, peripheral nerves and central nervous system, as well as orthopedics and trauma medicine. These pathologies are manifested by various clinical symptoms, including muscle weakness, change in muscle tone, co-activation patterns, as well as changes in the electromyography (EMG) signals. One such example is the change in activation of the first dorsal interosseous (FDI) muscle of the hand, resulting from lesions of the ulnar nerve (Kimura, 2013). In such cases, contemporary recordings rely on invasive needle EMG to reach single muscle specificity. Quantitative recordings from the FDI using printed electrode arrays can be readily achieved (Bareket et al., 2016; Inzelberg et al., 2018a). Another example is muscle atrophy, as in amyotrophic lateral sclerosis (ALS), where multiple muscles are recorded using needle EMG (Kimura, 2013). BSS is of importance in such an application as it can differentiate between muscle activations using a single integrated printed patch non-invasively (Figure 2D).

### The Future: Objective Physiological Evaluation for Everyday Clinics

With the increase of life expectancy, neurological disorders are becoming one of the greatest societal and economical challenges of our era. Despite the immensity of the problem, clinical evaluation still relies heavily on subjective judgment of an experienced physician. Patients who lack access to a high quality specialist evaluation may be undiagnosed or misdiagnosed. In both cases, they miss an opportunity for early diagnosis that can significantly affect disease management. Physicians also rely on patient self-reporting to assess symptoms that may not be apparent during a visit to the clinic. Moreover, symptom severity is almost impossible to quantify, therefore the efficacy of drugs is difficult to assess. Finally, latent clinical problems, which may already indicate disease onset, are very difficult to be identified. Printed electrodes on soft substrates together with advanced analysis schemes of the acquired data provide a simple and cheap tool for objective mapping of neurophysiological abnormalities. Additional sensors such as temperature and skin conductivity may further enhance the performances of printed films. Improving both printed technology and data analysis methodology, such as BSS (Urigüen and Garcia-Zapirain, 2015; Negro et al., 2016; Inzelberg et al., 2018b), classification (Debener et al., 2015; Zhang et al., 2016) and machine learning (Wang et al., 2014; Amin et al., 2015; Gokgoz and Subasi, 2015) will impact diagnosis, evaluation of treatment efficacy and enhance research of neuro-psychiatric disorders. Ideally, such systems will enable automatic feedback and screening of normal versus pathological performances.

## Summary

Printed electrodes can be used to perform high fidelity EEG and sEMG recordings and have countless applications in the medical and consumer markets. Further development may transform EEG and sEMG into a widely used diagnostic method in neurology, psychology and marketing. Further advancement of printed electronics may allow in the future the integration of additional capabilities beyond the sensing elements, such as amplifiers, temperature sensors, energy harvesting elements, chemical sensing and more.

## Ethics Statement

All experiments on human skin were conducted on volunteers in accordance with relevant guidelines and regulations under approval from the Institutional Ethics Committee Review Board at Tel Aviv University. Written informed consent was obtained from all subjects.

## Author Contributions

LI and YH wrote the manuscript. YH supervised the project.

## Conflict of Interest Statement

The authors declare financial interest in a new company (under formation) which will hold the licensing rights of the temporary-tattoo technology described in this paper.
